# Surgical outcomes of left hemicolon sparing resection versus extensive resection in treating synchronous colorectal cancer involving the right-sided colon and sigmoid colon or rectum

**DOI:** 10.1186/s12957-023-03012-x

**Published:** 2023-04-13

**Authors:** Jichuan Quan, Junguang Liu, Sicheng Zhou, Shiwen Mei, Wenlong Qiu, Yuanlian Wan, Xishan Wang, Jianqiang Tang

**Affiliations:** 1 Department of Colorectal Surgery, National Cancer Center/National Clinical Research Center for Cancer/Cancer Hospital, Chinese Academy of Medical Sciences and Peking Union Medical College, Beijing, 100021 China; 2 Department of General Surgery, Peking University First Hospital, Beijing, 100034 China

**Keywords:** Synchronous colorectal cancer, Extensive resection, Left hemicolon sparing, Anastomotic leakage, Prognosis

## Abstract

**Background:**

There are different surgical strategies that can treat synchronous colorectal cancer (SCRC) involving separate segments, namely extensive resection (EXT) and left hemicolon-sparing resection (LHS). We aim to comparatively analyze short-term surgical results, bowel function, and long-term oncological outcomes between SCRC patients treated with the two different surgical strategies.

**Methods:**

One hundred thirty-eight patients with SCRC lesions located in the right hemicolon and rectum or sigmoid colon were collected at the Cancer Hospital, Chinese Academy of Medical Sciences, and the Peking University First Hospital from January 2010 to August 2021 and divided into EXT group (*n* = 35) and LHS group (*n* = 103), depending on their surgical strategies. These two groups of patients were compared for postoperative complications, bowel function, the incidence of metachronous cancers, and prognosis.

**Results:**

The operative time for the LHS group was markedly shorter compared with the EXT group (268.6 vs. 316.9 min, *P* = 0.015). The post-surgery incidences of total Clavien-Dindo grade ≥ II complications and anastomotic leakage (AL) were 8.7 vs. 11.4% (*P* = 0.892) and 4.9 vs. 5.7% (*P* = 1.000) for the LHS and EXT groups, respectively. The mean number of daily bowel movements was significantly lower for the LHS group than for the EXT group (1.3 vs. 3.8, *P* < 0.001). The proportions of no low anterior resection syndrome (LARS), minor LARS, and major LARS for the LHS and EXT groups were 86.5 vs. 80.0%, 9.6 vs. 0%, and 3.8 vs. 20.0%, respectively (*P* = 0.037). No metachronous cancer was found in the residual left colon during the 51-month (median duration) follow-up period. The overall and disease-free survival rates at 5 years were 78.8% and 77.5% for the LHS group and 81.7% and 78.6% for the EXT group (*P* = 0.565, *P* = 0.712), respectively. Multivariate analysis further confirmed *N* stage, but not surgical strategy, as the risk factor that independently affected the patients’ survival.

**Conclusions:**

LHS appears to be a more appropriate surgical strategy for SCRC involving separate segments because it exhibited shorter operative time, no increase in the risk of AL and metachronous cancer, and no adverse long-term survival outcomes. More importantly, it could better retain bowel function and tended to reduce the severity of LARS and therefore improve the post-surgery life quality of SCRC patients.

## Introduction

The patient-related outcomes have become key factors to consider when deciding the most appropriate surgery strategy for patients with synchronous colorectal cancers (SCRC), especially those involving separate segments. In most cases, SCRC patients should be pre-surgically informed about the surgical risk and the surgery’s long-term impacts on their bowel habits and lifestyle, as well as whether they face the risk of developing another metachronous colon cancer lesion in the residual left hemicolon. Because the colon is critical for fluid absorption and fecal storage, extensive resection (EXT), such as subtotal colectomy or total colectomy, is expected to alter normal colon function and therefore lead to frequent and even disabling diarrhea [1]. Traditionally, patients with hereditary nonpolyposis colorectal cancer (HNPCC) or synchronous or metachronous cancers are more likely to be advised to undergo EXT rather than segmental resection because of EXT’s clinical advantages of eradicating synchronous pathology, preventing metachronous cancer, and reducing the need for complex surveillance [2–5].

For SCRC, how to select the most appropriate surgical plan is still a problem faced by surgeons [1, 5–10]. For cases with one lesion localized in the right hemicolon and the other in the rectum or sigmoid colon, the selection between left hemicolon sparing (LHS) with double anastomoses and EXT, such as subtotal colectomy, total colectomy, or proctocolectomy with ileoanal anastomosis, is still controversial. Whether LHS affects the surgical outcomes of SCRC patients, especially the risk of anastomotic leakage (AL), is currently understudied. Balancing the tradeoffs between a potentially better bowel function against an increased risk of AL of SCRC patients following LHS requires specific knowledge on both surgical risks and the degree of functional compromise in relevant patient populations.

Therefore, in the present study, we undertook a retrospective two-institution research to compare the surgical outcomes of patients treated with LHS and EXT for SCRC involving separated segments.

## Material and methods

### Study population

We included SCRC patients who underwent surgical treatment at the Cancer Hospital, Chinese Academy of Medical Sciences, and the First Hospital of Peking University from January 2010 to August 2021. The diagnosis of multiple colorectal cancer (CRC) lesions was established based on the criteria reported by Warren and Gates [11]. Inclusion criteria are as follows: (1) SCRC patients whose lesions were pathologically confirmed as primary adenocarcinoma and (2) SCRC patients with one lesion in the right hemicolon and the other in the rectum or sigmoid colon. The exclusion criteria included are as follows: (1) Patients with ulcerative colitis (UC), familial adenomatous polyposis (FAP), HNPCC, or Lynch Syndrome (LS); (2) patients with SCRC involving the same segment or adjacent segments; (3) SCRC patients with distant metastases; and (4) SCRC patients who underwent Hartmann or abdominal perineal resections. Patients were categorized into two groups based on the surgical method, namely an LHS group and an EXT group. The surgical outcomes, postoperative complications, postoperative defecation function, low anterior resection syndrome (LARS), and oncological outcomes were comparatively analyzed for SCRC patients treated with the two different surgical strategies. The ethics committees of the participating institutions granted approval for this research.

### Data collection

Data on patients’ clinicopathological characteristics, including preoperative variables, intraoperative variables, and postoperative variables, were collected. The preoperative variables considered included age (two age groups [≤ 63 years and > 63 years] were generated according to the patients’ mean age at diagnosis), gender, body mass index (BMI), abdominal surgery history, concomitant diseases, preoperative chemotherapy, hemoglobin (Hb) level, serum albumin (Alb) level, carbohydrate antigen 19–9 (CA199) level, carcinoembryonic antigen (CEA) level, and American Society of Anesthesiologists (ASA) physical status. The intraoperative variables included operative approach (laparoscopic/open), operative time, volume of blood loss, and type of surgical resection (LHS or EXT). The postoperative variables included length of postoperative hospital stay, postoperative complications, classification of complications, mortality, re-operation, number of daily bowel movements, LARS score, tumor size, tumor differentiation status, N stage, T stage, and TNM stage. Patients were followed up by telephone call or outpatient examination. To comparatively analyze postoperative bowel function and oncological outcomes between the LHS and EXT groups, data on postoperative defecation function, postoperative incidence of metachronous CRC, disease-free survival (DFS), and overall survival (OS) were collected. Grading of postoperative complications was performed as the Clavien-Dindo (CD) classification [12], and postoperative defecation function was appraised by LARS scores [13], and was investigated at the median time of 51 months after the surgery. In this study, the duration from the surgery date to the date of death represented OS, while that from the surgery date to the date on which tumor recurrence or distant metastasis was diagnosed represented DFS.

### Surgical procedures

**Fig. 1:**
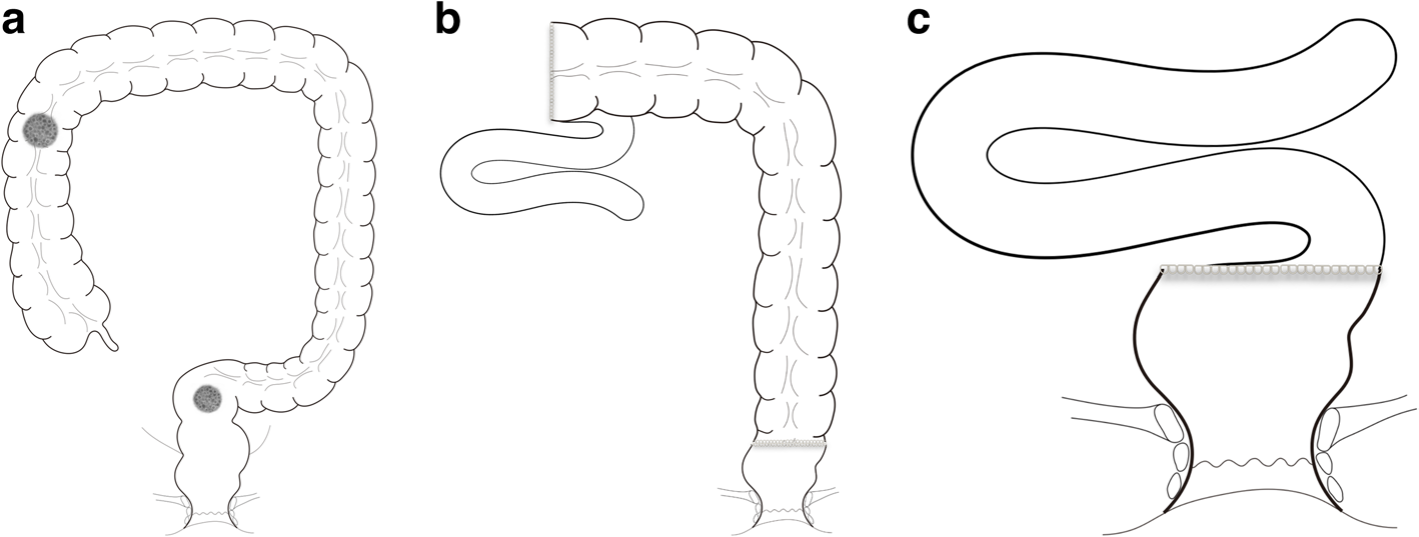
Pattern diagram of different surgical methods. **a** Diagram of a separate segment; **b** Diagram of left hemicolon sparing; **c** Diagram of extensive resection

Either laparoscopic surgery or open surgery was performed by experienced surgeons. For patients in the LHS group, the patients underwent right hemicolectomy and anterior resection of the rectum or right hemicolectomy and sigmoid colectomy. The procedure for conventional right hemicolectomy, including mobilization of the right colon, ligation of the ileocolic vessels, right colic vessels, and right branch of middle colic vessels, dissection of draining lymph nodes, and ileocolonic anastomosis, was performed. For lesions localized in the rectal or sigmoid colon, the corresponding section was mobilized; the inferior mesenteric vessels were ligated with or without left colonic artery preservation; draining lymph nodes were dissected according to the standard procedure; and finally, sigmoid-rectal anastomosis was performed by staplers. For patients in the EXT group, right hemicolon and left hemicolon or rectum were mobilized; the ileocolic vessels, right and middle colic vessels, and inferior mesenteric vessels were ligated; draining lymph nodes were dissected according to the standard procedure; and finally, total colectomy, subtotal colectomy, or proctocolectomy was finished with ileo-sigmoid/ileo-rectal/ileo-anal anastomosis by staplers. Pattern diagrams of the two surgical methods are shown in Fig. 1a–c.

### Follow-up

Patients were followed up by telephone call or outpatient examination. Follow-up was conducted every 3 months for the first 2 years, every 6 months for 3–5 years, and annually after 5 years. Follow-up included physical examination, serum tumor markers, CT examinations of the chest, abdomen, and pelvic, and colonoscopy. For colonoscopy examination, our standard postoperative surveillance was to perform the 1st colonoscopy examination at 1 year after surgery; for patients with polyps before surgery, the 1st colonoscopy was performed to remove the polyps at 3 months after surgery. Thereafter, a colonoscopy was performed once a year.

### Statistical analysis

Categorical and continuous variables were respectively analyzed by chi-square/Fisher’s exact and Student’s *t*/Mann–Whitney *U* tests. Kaplan–Meier survival curves were generated, based on which log-rank tests were performed to compare survival differences between the two groups of patients. Cox proportional hazards models were applied to perform univariate and multivariate analyses of prognostic factors. Two-sided *P* values less than 0.05 signify statistical significance. All statistical analyses were implemented with SPSS version 20.0 (IBM, Armonk, NY, USA).

## Results

### Basic characteristics of the selected patients

**Fig. 2:**
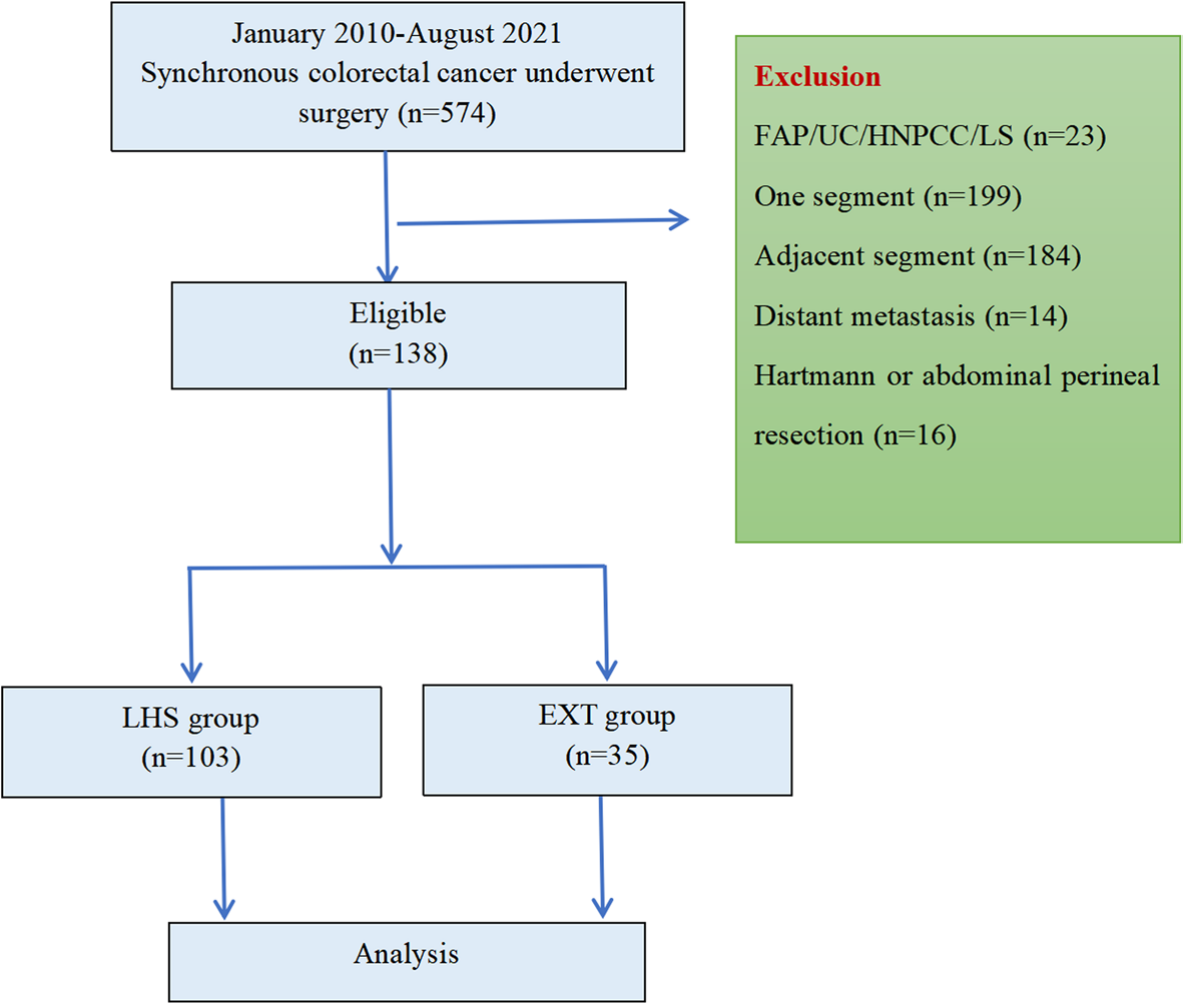
Patient selection

SCRC patients treated with surgeries in the two institutions from January 2010 to August 2021 were recruited, yielding an initial cohort of 574 patients. Among them, 436 patients were excluded, including 23 cases with UC, FAP, HNPCC, or LS, 199 cases with SCRC lesions located in the same segment, 184 cases with SCRC lesions located in adjacent segments, and 30 cases with SCRC lesions located in separate segments but underwent Hartmann/abdominal perineal resections or with distant metastasis. Ultimately, 138 SCRC patients were enrolled, among which 103 were assigned to the LHS group and 35 were assigned to the EXT group (Fig. 2).

The basic characteristics of the two groups of patients are displayed in Table 1. We did not observe any significant differences in age, gender, BMI, history of abdominal surgery, concomitant diseases, preoperative chemotherapy, Hb level, serum Alb level, CA199 level, CEA level, ASA physical status, the distances from rectal or sigmoid colon cancer lesions to the anal verge, tumor differentiation status, tumor size, pT stage, pN stage, and TNM stage between the two groups.

**Table 1:** Basic characteristics between left hemicolon sparing and extensive resection

Variable	LHS group (*n* = 103)	EXT group (*n* = 35)	*P*
Age, (years)			
≤ 63	44 (42.7)	20 (57.1)	0.139
> 63	59 (57.3)	15 (42.9)	
Gender			
Female	38 (36.9)	10 (28.6)	0.372
Male	65 (63.1)	25 (71.4)	
BMI, mean ± SD, kg/m^2^	23.8 ± 3.4	24.8 ± 3.0	0.145
ASA physical status			
I–II	90 (87.4)	34 (97.1)	0.184
III	13 (12.6)	1 (2.9)	
Concomitant diseases			
No	45 (43.7)	15 (42.9)	0.932
Yes	58 (56.3)	20 (57.1)	
History of abdominal surgery			
No	81 (78.6)	26 (74.3)	0.594
Yes	22 (21.4)	9 (25.7)	
Preoperative chemotherapy			
No	101 (98.1)	35 (100.0)	1.000
Yes	2 (1.9%)	0 (0.0)	
The distance of rectal or sigmoid colon cancer from anal verge, cm	12 (3–30)	15 (3–32)	0.294
Tumor size^a^, cm			
≤ 5	56 (54.9)	18 (51.4)	0.722
> 5	46 (45.1)	17 (48.6)	
Tumor differentiation			
Well-moderate	53 (51.5)	21 (60.0)	0.381
Poor	50 (48.5)	14 (40.0)	
pT stage			
T1-T2	8 (7.8)	5 (14.3)	0.254
T3-T4	95 (92.2)	30 (85.7)	
pN stage			
N0	41 (39.8)	14 (40.0)	0.933
N1	44 (42.7)	14 (40.0)	
N2	18 (17.5)	7 (20.0)	
Stage			
I	7 (6.8)	4 (11.4)	0.619
II	34 (33.0)	10 (28.6)	
III	62 (60.2)	21 (60.0)	
Hb level			
< 120	49 (47.6)	16 (45.7)	0.944
≥ 120	36 (35.0)	12 (34.3)	
Unknown	18 (17.5)	7 (20.0)	
Alb level			
< 40	50 (48.5)	13 (37.1)	0.312
≥ 40	35 (34.0)	12 (34.3)	
Unknown	18 (17.5)	10 (28.6)	
CEA level			
≤ 5	45 (43.7)	11 (31.4)	0.441
> 5	32 (31.1)	13 (37.1)	
Unknown	26 (25.2)	11 (31.4)	
CA19-9 level			
≤ 37	64 (62.1)	20 (57.1)	0.881
> 37	12 (11.7)	4 (11.4)	
Unknown	27 (26.2)	11 (31.4)	

*Abbreviation: LHS* left hemicolon sparing, *EXT* extensive resection, *BMI* body mass index, *Hb* hemoglobin, *Alb* albumin, *CA19-9* carbohydrate antigen 19–9, *CEA* carcinoembryonic antigen, *ASA* American Society of Anesthesiologists

^a^Unknown for one patient

### Surgical results and bowel function data

Surgical results and bowel function data for patients in both groups are shown in Table 2. The operative time for the LHS group was markedly shorter compared with the EXT group (268.6 vs. 316.9 min, *P* = 0.015). The two groups’ operative approach, blood loss volume, and duration of post-surgery hospital stay did not differ significantly. The post-surgery incidence of CD grade ≥ II total complications was 8.7% and 11.4% for the LHS and EXT groups, respectively. Between the two groups, no statistically significant differences existed in mortality and the incidences of ileus, AL, and abdominal incision infection. In the LHS group, five patients with double anastomoses were diagnosed with postoperative AL, including four cases of sigmoid-rectal AL and one case of ileocolonic AL; while in the EXT group, ileo-rectal AL was confirmed in two patients. Of 7 cases with AL, 5 patients underwent re-operation.

**Table 2:** Surgical results and bowel function analysis between left hemicolon sparing and extensive resection

Variable	LHS group (*n* = 103)	EXT group (*n* = 35)	*P*
Operative type			
Open	41 (39.8)	14 (40.0)	0.984
Laparoscopic	62 (60.2)	21 (60.0)	
Operative time, mean ± SD, min	268.6 ± 92.2	316.9 ± 120.7	0.015
Blood loss, median, range, mL	100 (20–600)	100 (30–600)	0.506
Postoperative complications (Grade II-V)	9 (8.7)	4 (11.4)	0.892
Ileus	2 (1.9)	1 (2.9)	1.000
Anastomotic leakage	5 (4.9)	2 (5.7)	1.000
Sigmoid-rectal	4 (3.9)	-	
Ileo-colonic	1 (1.0)	-	
Ileo-sigmoid/ileo-rectal	-	2 (5.7)	
Cerebral infarction	1 (1.0)	0 (0.0)	1.000
Abdominal incision infection	1 (1.0)	1 (2.9)	0.444
Re-operation	4 (3.9)	1 (2.9)	1.000
Mortality	0 (0)	0 (0)	1.000
Postoperative hospital stay, median, range, days	10 (3–34)	11 (6–38)	0.101
Metachronous colorectal cancer	0 (0)	0 (0)	1.000
Anal function			
LARS score			0.037
No LARS	45 (86.5)	16 (80.0)	
Minor LARS	5 (9.6)	0 (0.0)	
Major LARS	2 (3.8)	4 (20.0)	
Number of daily bowel movements (mean ± SD)	1.3 ± 0.6	3.8 ± 2.3	0.000

*Abbreviation: LHS* left hemicolon sparing, *EXT* extensive resection, *LARS* low anterior resection syndrome

As for postoperative defecation function, the mean number of daily bowel movements for the LHS group was markedly lower compared with the EXT group (1.3 vs. 3.8, *P* < 0.001). In addition, according to LARS scores, 9.6% and 3.8% of patients in the LHS group developed minor and major LARS, respectively, whereas the proportions of no LARS, minor LARS, and major LARS for patients for the EXT group were 80%, 0%, and 20%, respectively. Therefore, the proportion of patients who developed postoperative major LARS was significantly higher in the EXT group than in the LHS group (*P* = 0.037).

### Long-term oncological outcomes and survival analysis results

**Fig. 3:**
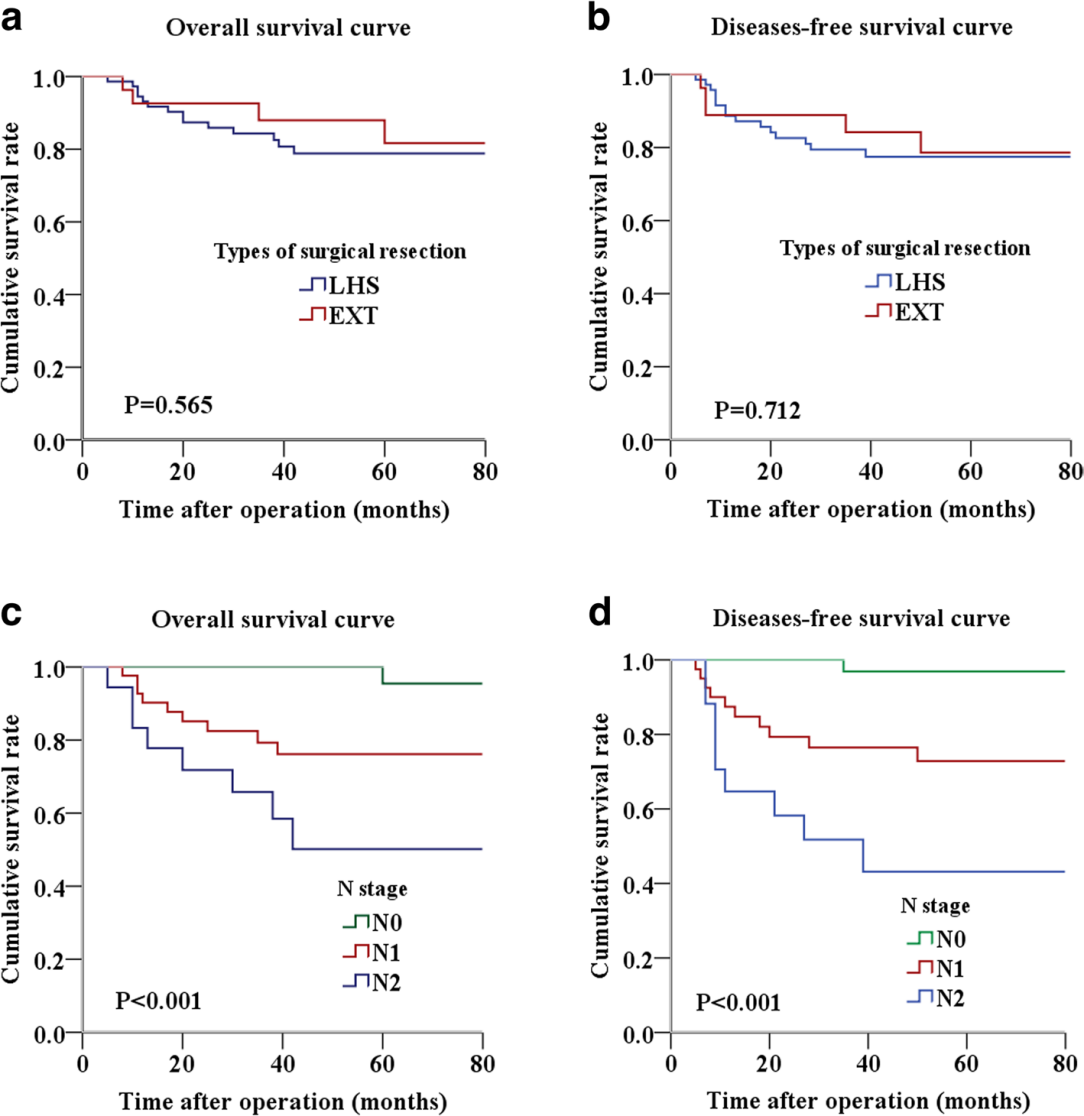
Kaplan–Meier survival analysis. **a** Overall survival curves for patients with different surgical methods; **b** diseases-free survival curves for patients with different surgical methods; **c** overall survival curves for patients with different *N* stage; **d** diseases-free survival curves for patients with different *N* stage

The median follow-up time for the patients was 51 months. During the follow-up, no metachronous cancer was found in the residual left colon for the LHS group. The OS at 1, 3, and 5 years was 93.1%, 84.3%, and 78.8% for the LHS group and 92.6%, 88.0%, and 81.7% for the EXT group, respectively, while the DFS at 1, 3, and 5 years was 88.6%, 79.5%, and 77.5% for the LHS group and 88.9%, 84.2%, and 78.6% for the EXT group, respectively. We observed no significant differences in OS (*P* = 0.565) and DFS (*P* = 0.712) between the two groups (Fig. 3a, b).

Factors that affected the survival of these SCRC patients were explored via univariate and multivariate prognostic analyses. The data indicated that the *N* stage was a significant risk factor influencing OS and DFS (Table 3). According to the established survival curves, patients with N0-stage SCRC had markedly longer OS and DFS (both *P* values < 0.001) than those with N1- and N2-stage SCRC for both groups (Fig. 3c, d).

**Table 3:** Univariate and multivariate analysis of overall survival and disease-free survival

Variables	Overall survival				Disease-free survival			
	Univariable analysis		Multivariate analysis		Univariable analysis		Multivariate analysis	
	HR (95% CI)	*P* value	HR (95% CI)	*P* value	HR (95% CI)	*P* value	HR (95% CI)	*P* value
Age (> 63/ ≤ 63 years)	2.384 (0.914–6.219)	0.076			1.418 (0.597–3.369)	0.428		
Gender (male/female)	0.723 (0.299–1.748)	0.472			0.880 (0.365–2.125)	0.777		
Hb level (≥ 120/ < 120)	1.520 (0.616–3.749)	0.363			2.133 (0.858–5.303)	0.103		
Alb level (≥ 40/ < 40)	1.222 (0.439–3.406)	0.701			1.472 (0.530–4.084)	0.458		
CEA level (> 5/ ≤ 5)	1.143 (0.377–3.467)	0.813			0.866 (0.300–2.500)	0.790		
CA19-9 level (> 37/ ≤ 37)	1.554 (0.425–5.680)	0.505			0.902 (0.202–4.036)	0.893		
ASA physical status (III/I-II)	2.376 (0.785–7.190)	0.126			2.049 (0.602–6.977)	0.251		
Operative approach (laparoscopy/open)	1.658 (0.626–4.394)	0.309			1.278 (0.511–3.199)	0.600		
Type of surgical resection (EXT/LHS)	0.725 (0.240–2.188)	0.568	0.836 (0.275–2.543)	0.752	0.828 (0.303–2.267)	0.713	0.836 (0.305–2.292)	0.727
Tumor differentiation (poor/well- moderate)	1.721 (0.711–4.166)	0.228			1.692 (0.709–4.037)	0.236		
Tumor size (> 5/ ≤ 5 cm)	1.271 (0.516–3.132)	0.602			0.899 (0.378–2.139)	0.811		
T stage (T3-T4/T1-T2)	1.672 (0.222–12.583)	0.618			1.880 (0.251–14.055)	0.539		
*N* stage								
N0	-	-	-	-	-	-	-	-
N1	10.952 (1.401–85.584)	0.023	10.899 (1.394–85.190)	0.023	13.462 (1.736–104.358)	0.013	13.461 (1.736–104.353)	0.013
N2	29.321 (3.699–232.448)	0.001	28.923 (3.643–229.610)	0.001	32.330 (4.067–256.983)	0.001	32.288 (4.062–256.630)	0.001

*Abbreviation: LHS* left hemicolon sparing, *EXT* extensive resection, *Hb* hemoglobin, *Alb* albumin, *CA19-9* carbohydrate antigen 19–9, *CEA* carcinoembryonic antigen, *ASA* American Society of Anesthesiolog, *HR* hazard ratio, *CI* confidence interval

## Discussion

Previous studies comparing the outcomes of segmental resection and extensive resection strategies for SCRC patients have exhibited comparable rates of grades II and III postoperative morbidities [14]. In addition, patients subjected to EXT may experience more severely compromised bowel function and life quality, even after long-term adaptation [1, 14]. However, for patients who are diagnosed with SCRC at a young age, those with a strong familial inheritance of the disease, those with multiple/metachronous lesions, or those who are more genetically susceptible to cancers, EXT may represent a better option than segmental resection [4, 6]. Therefore, it is crucial to compare the outcomes of EXT and segmental resection in various SCRC cohorts to enrich our knowledge on the two surgical strategies, thereby guiding the clinical treatment of the disease.

Unlike previous reports [15–24], we recruited a cohort of SCRC patients whose lesions were located in separate segments and compared the differences between LHS and EXT. Our study showed that LHS did not increase the incidences of surgical complications and AL compared with EXT. In addition, OS, DFS, and the incidence of metachronous colorectal cancers were all similar between the two groups. However, the LHS group tended to have a decreased number of daily bowel movements and a lower percentage of major LARS cases compared with the EXT group. Multivariate analysis further confirmed the *N* stage as the only factor that could independently predict OS and DFS in SCRC cases involving separate segments, whereas surgical strategy had no significant impact on the patients’ survival. Ours is the research comprehensive comparing the surgical results, incidence of metachronous colorectal cancer, and long-term oncological outcomes between LHS and EXT for non-HNPCC patients with SCRC involving separate segments based on a relatively large SCRC cohort. More importantly, we analyzed the impact of a surgical method on functional sequelae (in terms of defecation function and LARS) in this cohort.

LHS involves two anastomoses, namely an ileocolonic anastomosis and a colorectal anastomosis. The preserved left branch of the middle colon artery, left colonic artery, and inferior mesenteric artery can ensure blood supply in the residual left hemicolon. However, the risk of ischemia in the left hemicolon will be increased if the operator is inexperienced with the ligation of key blood vessels. At present, there are few studies on whether the ileocolonic anastomosis will affect the safety of colorectal anastomosis. Holubar et al. stood the point that synchronous double colon anastomoses may not increase the risk of developing complications and are a safe regimen for certain patients, and there were no anastomotic leaks or fistulas in 69 patients subjected to double colonic anastomoses [7]. In a previous study, Takatsu et al. retrospectively analyzed 42 patients who underwent laparoscopic (*n* = 27) and open (*n* = 15) double colon resections and anastomoses, the AL rate was 9.5% (3.7% and 20.0% in the laparoscopic and open surgery groups, respectively), and these data indicate that laparoscopic surgeries for SCRC are safe and have more short-term benefits than open surgeries [25]. The overall AL rate in our cohort was 5.1%, and the AL rate was 4.9% for the LHS group, similar to the rate of ileorectal/ileosigmoid AL (5.7%) for the EXT subgroup. As for operative time, it should be noted that although an additional anastomosis was performed for the LHS group, the mean operative time for this group was 48 min shorter than that for the EXT group, which may be ascribed to the dissociation of left hemicolon and the use of ileal pouch during the EXT procedure.

We also investigated whether LHS will increase the incidence of metachronous colorectal cancer in the left hemicolon or affect the radicality. Our results showed that the 103 patients in the LHS group did not develop metachronous cancer during the long-term follow-up. At the same time, OS and DFS exhibited no significant differences between the LHS and EXT groups. Our study excluded cases with FAP and regularly performed colonoscopy examinations for patients. Our standard postoperative surveillance was to perform the 1st colonoscopy examination at 1 year after surgery; for patients with polyps before surgery, the 1st colonoscopy was performed to remove the polyps at 3 months after surgery. Thereafter, a colonoscopy was performed once a year. In this way, precancerous lesions were removed to prevent them from developing into cancer lesions. Therefore, for our cohort, there was no re-operation due to metachronous cancers during the follow-up. We further analyzed the prognostic factors for SCRC involving separate segments within a median follow-up duration of 51 months. The results confirmed that lymph node metastasis status could independently affect both OS and DFS for SCRC involving separate segments, while the surgical strategy (LHS or EXT) did not significantly affect the patients’ OS and DFS.

The colon plays key physiological roles in defecation and maintaining fluid balance. Patients who are about to undergo colorectal operations often expect an altered bowel function and thus seek counseling preoperatively. A previous study [14], which was designed to predict postoperative functional outcomes and quality of life after colonic resections of different lengths, demonstrated that the patients had to contend with frequent stools after the surgeries. In addition, the concurrent analyses of the study also indicated restricted preoperative social activity, housework, recreation, and travel due to the altered bowel function after EXT. In our study, the mean numbers of daily bowel movements were 3.8 in the EXT group (similar to that reported in the study mentioned above) and 1.3 in the LHS group. In this study, we also utilized the LARS score to compare surgical outcomes of LHS and EXT. Our findings indicated that patients with major LARS scores in the LHS group were less than those in the EXT group. These findings further confirmed the benefits of preserving the left hemicolon on bowel function in SCRC cases involving separate segments.

Our study has several limitations that ought to be mentioned. First, our study has inherent limitations due to the retrospective nature and the relatively small number of participants. Second, the bowel function scale was partially lost, and the quality of life was not scored. In addition, some of the patients may not have achieved stable functional outcomes at the time of the survey. Third, the follow-up data were incomplete, and some variables such as the occurrence of benign polyps in the left hemicolon were not described in detail. In view of these limitations, our conclusions warrant further validation by prospective studies that include more participants, adopt more detailed and longer follow-up, and perform more objective bowel functional assays. Nevertheless, so far as we know, we recruited the largest cohort of SCRC patients reported so far to confirm that LHS has acceptable surgical safety and AL rate, better preserves bowel function, and possesses similar long-term oncological outcomes as compared with EXT.

## Conclusions

Our study proposes that LHS appears to be a more appropriate surgical strategy for treating SCRC involving separate segments patients who were non-HNPCC, because it has the advantages of acceptable surgical safety, no increase in AL rate, an extremely low risk of developing metachronous cancer, and no adverse long-term survival outcomes. More importantly, it will better retain bowel function and alleviate LARS severity, thereby ameliorating the life quality of patients with SCRC involving separate segments. Therefore, it is reasonable to speculate that the traditional extensive resection method for surgical removal of SCRC involving separate segments, which generally leads to an increased number of daily bowel movements and a decline in the quality of life, will face a greater possibility of being replaced by segmental resection strategies in the future.

## Data Availability

The datasets used and/or analyzed during the current study are available from the corresponding author on reasonable request.

## Abbreviations

SCRC: Synchronous colorectal cancer
EXT: Extensive resection
LHS: Left hemicolon sparing resection
AL: Anastomotic leakage
LARS: Low anterior resection syndrome
HNPCC: Hereditary nonpolyposis colorectal cancer
UC: Ulcerative colitis
LS: Lynch syndrome
CRC: Colorectal cancer
FAP: Familial adenomatous polyposis
BMI: Body mass index
Hb: Hemoglobin
Alb: Albumin
CA19-9: Carbohydrate antigen 19–9
CEA: Carcinoembryonic antigen
ASA: American Society of Anesthesiologists
DFS: Disease-free survival
OS: Overall survival
CD: Clavien-Dindo
SPSS: Statistical Product and Service Solutions
